# Essential Oil and Plant Extracts as Preservatives and Natural Antioxidants Applied to Meat and Meat Products: A Review

**DOI:** 10.17113/ftb.61.02.23.7883

**Published:** 2023-06

**Authors:** Gabriela Aguiar Campolina, Maria das Graças Cardoso, Alex Rodrigues-Silva-Caetano, David Lee Nelson, Eduardo Mendes Ramos

**Affiliations:** 1 Food Sciences Department, Federal University of Lavras (UFLA), Professor Edmir Sá Santos Rotatory Clover, 3037 Lavras, MG, Brazil; 2 Chemistry Department, Federal University of Lavras (UFLA), Professor Edmir Sá Santos Rotatory Clover, 3037 Lavras, MG, Brazil; 3 Postgraduate Program in Biofuels, Federal University of the Jequitinhonha and Mucuri Valleys, MGT 367 Highway Km 583, N/N Diamantina, MG, Brazil

**Keywords:** natural compounds, antimicrobial activity, antioxidant activity, meat industry, essential oils, plant extracts

## Abstract

The meat and meat product industry has evolved according to the needs of the market. Consumers are increasingly seeking quality in food. Thus, the concern regarding the excessive use of additives such as preservatives and antioxidants has driven research towards natural, healthy and safe substitutes. Essential oils and plant extracts have been shown to be a good option for resolving this problem. They are completely natural with biological activity, which mainly includes prevention of oxidation and the proliferation of microorganisms, thus arousing the interest of the industry and consumers. This review will present studies published in the last five years regarding the potential of essential oils and plant extracts to act as preservatives and antioxidants in meat and meat products. The forms of application, innovations in the area, alternatives to the incorporation of essential oils and extracts in meat products, effects caused in food, and limitations of applications will be detailed and discussed.

## INTRODUCTION

Meat can be defined as animal tissue that is suitable for human consumption. Like meat, its products are complex, highly perishable foods and have in their composition, in addition to proteins, saturated and unsaturated lipids, carbohydrates, vitamins and pigments that can undergo oxidation reactions and microbial deterioration. Thus, the shelf life of meat is influenced by several factors, such as storage temperature, enzyme action, oxygen, humidity, light and microorganisms. The influence of these factors is worrisome because they directly interfere with the quality of food, both nutritionally and in sensory aspects. They can cause changes in attributes such as texture, colour, odour, flavour and aroma (*1*, *2*).

Oxidation is a process that frequently occurs in meat during storage. The oxidation of lipids, proteins and pigments directly interferes with the sensory and nutritional quality of the product. In addition, toxic compounds can be produced (*1*, *3*). Oxidation is a factor that must be controlled in meat. However, the proliferation of microorganisms is a factor that deserves even more attention because of the harm they can cause to consumers.

The contaminating and spoilage microorganisms in meat are mostly pathogenic bacteria *Campylobacter* spp., *Listeria monocytogenes*, *Staphylococcus aureus, Salmonella enterica* and *Escherichia coli*, which are responsible for foodborne outbreaks. The bacterial genera that deserve attention are *Acinetobacter, Alteromonas, Aeromonas, Brochothris, Flavobacterium, Leuconostoc, Pseudomonas, Moraxella,* lactic acid bacteria and those belonging to the *Enterobacteriaceae* family (*4*, *5*).

The use of food additives, such as preservatives and antioxidants, has been of global concern in recent years. One of the foods that generate greater concern regarding the use of additives is meat and its derived products. Originating from cattle, swine or poultry, meat and meat products are highly perishable, susceptible to the action of various microorganisms and lipid oxidation. Therefore, methods to maintain quality and increase their shelf life are required, one of the methods being the addition of antioxidants and preservatives.

The food industry has constantly sought ways to minimize the loss of quality in meat and its products and increase its shelf life. Conservation methods such as low temperature, specific packaging and adequate storage are frequently used. In addition, the use of additives that act as preservatives and antioxidants is often essential to ensure the quality of meat and its products. However, the use of synthetic additives can be harmful to health, and this fact is increasingly noticeable to consumers, who are searching for healthy foods that are more natural and prefer natural compounds (*6*). According to Lin and Wu (*6*), the fact that plants are the main natural source of antioxidants and, in general, do not pose risks to food safety, makes the use of plant derivatives as antioxidants valuable. Economic growth and the emergence of new technologies have also increased the demand for natural products. Thus, the application of compounds such as essential oils and plant extracts that act as natural preservatives emerges as an interesting strategy to reduce or replace the use of traditional synthetic additives if they are equally efficient (*6*, *7*).

This review will address some alternatives researched in the last five years for the application of essential oils and plant extracts in meat and meat products with the aim of preserving and replacing, in whole or in part, synthetic preservatives and antioxidants generally applied to these foods. In addition, the mechanisms of antioxidant and antimicrobial action of the natural products under study will be briefly discussed.

## ESSENTIAL OILS AND PLANT EXTRACTS

Essential oils are volatile organic compounds synthesized by plants in response to physiological stress, ecological factors and pathogen attack, as well as acting to attract pollinators to facilitate reproduction (*8*). They can be defined as ’the product obtained from a natural raw material of vegetable origin by steam distillation, from the epicarp of citrus fruits by mechanical processes or by dry distillation, after separation of the aqueous phase, if any, by physical processes’. ISO 9235:2021 (*9*) also emphasizes that steam distillation can be performed with the addition of water to the distillate, a process known as hydrodistillation.

The main characteristics of the essential oils are their complex compositions of low-molecular-mass molecules with different chemical structures that include monoterpenes, sesquiterpenes, alcohols, aldehydes, esters, ethers, ketones, various phenylpropanoid derivatives and various volatile organic compounds. In addition, they are liquid at room temperature and hydrophobic so they have low water solubility (*10*, *11*).

Vegetable extracts, unlike essential oils, are preparations obtained by the extraction of the active constituents of vegetables and must contain sapid, aromatic, volatile and fixed properties corresponding to the respective natural product. The active ingredients can be extracted using different solvents such as methanol, ethyl acetate, hexane, ethanol, or acetone, and the material used in the extraction can be previously treated by means of enzymatic inactivation, milling or degreasing. After extraction, undesirable compounds can also be eliminated by purifying the extract (*12*, *13*).

Essential oils and plant extracts, in addition to being natural products extracted from plants, are mostly considered to be GRAS (Generally Recognized as Safe), which allows their use in food products without posing risks to consumers. In addition, different biological activities can be attributed to them, including antioxidant and antimicrobial activity, depending on their compositions. Phenolic compounds, alcohols, aldehydes, phenylpropanoids, terpenes and ketones are the principal constituents responsible for the antioxidant activity of essential oils. They protect against pro-oxidants naturally present in meat, such as free iron ions (*1*). In terms of antimicrobial activity, the constituents that stand out are those containing aromatic oxygen compounds with carbonyl groups (aldehydes and ketones), phenols, ethers or acids, followed by oxygenated aliphatic terpenes (*14*).

Plant extracts also contain phytochemicals of interest. Those that stand out, such as phenolic compounds, have antioxidant and antimicrobial activities. In particular, there are the tannins and flavonoids that can be subclassified into flavones, flavanones, flavonols, flavanonols, isoflavones, catechins and anthocyanidins (*15*, *16*).

## MECHANISM OF ANTIMICROBIAL ACTIVITY OF ESSENTIAL OILS AND PLANT EXTRACTS

Psychrotrophic *Pseudomonas*, lactic acid bacteria, *Enterobacteriaceae* and *Clostridium* spp. are one of the principal spoilage groups in freshly stored and refrigerated meat because they have the ability to be developed at temperatures below 7 °C. The activity of essential oils and plant extracts against microorganisms is directly related to their constituents. However, it is worth noting that the combination of constituents can act synergistically in the antimicrobial mechanism (*17*-*19*).

**Fig. 1:**
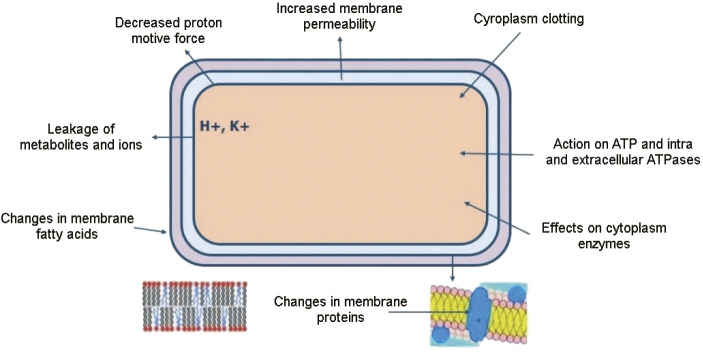
Possible cellular targets of antibacterial action by natural compounds

The principal mechanism of action of both essential oils and plant extracts involves the interaction with the cell membrane of microorganisms (Fig. 1). These natural compounds can act by increasing membrane permeability, inhibiting the absorption of substrates that are important for microbial growth, and interfering with the cellular metabolism (*20*, *21*).

Studies report that Gram-positive bacteria are more susceptible to the action of the constituents. This observation can be explained by the involvement of the lipopolysaccharide layer present in the cell wall of Gram-negative bacteria. This layer limits the diffusion of hydrophobic compounds, such as essential oils (*22*).

Thus, the mechanism of action of the essential oils involves the interaction of their constituents with the cell membranes of microorganisms, which are composed of lipids. The cell membrane, when interacting with constituents, can be damaged, leading to an increase in membrane permeability and impairment of functions in the cell such as nutrient uptake, electron transport, nucleic acid synthesis, enzyme activity and can even cause death. In addition, the constituent molecules of essential oils can cross the membrane and reach the cytoplasm, where they can react with other cellular components (*4*, *23*).

## MECHANISM OF ANTIOXIDANT ACTIVITY OF ESSENTIAL OILS AND PLANT EXTRACTS

The oxidation of lipids present in meat leads to the formation of hydroperoxides, which in turn generate degradation products in meat and compounds such as volatile and undesirable aldehydes, ketones, acids and alcohols. Protein oxidation causes changes in proteins and amino acids. Thus, the level of digestibility, solubility and bioavailability can be reduced. Pigments, such as myoglobin, which is one of the main pigments responsible for the colour of the product, form brown compounds when oxidized and thus affect the appearance of the meat (*24*, *25*).

**Fig. 2:**
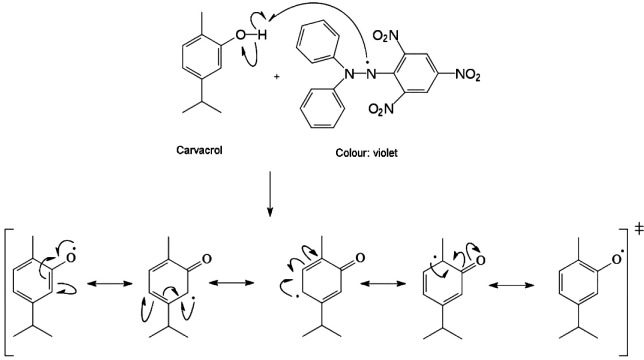
Antioxidant action of carvacrol by transfering a hydrogen atom from the OH group

The phenolic compounds present in plant extracts are considered to be the main group responsible for the antioxidant activity of the extracts. In essential oils, phenylpropanoids and terpenoids with phenolic characteristics also have antioxidant activities. These compounds can act in the stabilization of free radicals because their structures bear a hydroxyl group (-OH) on a benzene ring. Thus, they can act by transferring the H atom from the OH group to the free radical, as reducing agents and singlet oxygen inhibitors, as shown in Fig. 2 (*21*, *26*, *27*).

## APPLICATIONS OF ESSENTIAL OILS AND PLANT EXTRACTS AS PRESERVATIVES AND ANTIOXIDANTS IN FRESH MEAT AND MEAT PRODUCTS

The proliferation of microorganisms can cause deterioration and contamination of the product, making its commercialization and consumption unfeasible. Food industries, in general, add antioxidants and preservatives to meat products to preserve their microbiological, physicochemical and sensory characteristics. Thus, the application of essential oils and plant extracts to meat products has been shown to be a natural and efficient alternative to preserve these products, preventing the proliferation and action of microorganisms.

The application of natural compounds such as essential oil and plant extracts in meat products basically boils down to the direct application to the meat product, whether diluted or not, and application through nanoemulsions, nanoparticles and active and intelligent packaging, such as films and coatings. In all the forms of application, a combination with other conservation methods, such as refrigeration, freezing and appropriate packaging, is necessary.

### Application of essential oils and plant extracts directly on meat and meat products: preservative and antioxidant activity

Some of the main studies of the direct application of essential oil and plant extracts to meat and meat products are presented in Table 1 (*22*, *28*-*32*) and Table 2 (*22*, *33*-*41*), respectively. Danilović *et al*. (*22*) evaluated the application of essential oils and sage extract to pork to control *E. coli*. Pork pieces were treated separately with essential oil and extract. The results showed that, after 14 days, a significant inhibition of *E. coli* growth was observed in the treatments involving the addition of the essential oil at all tested concentrations. The treatment with sage extract had a smaller effect against the evaluated microorganism than the treatment with the essential oil, but the proliferation of the bacteria decreased to a concentration of 1.0 µL/g. However, regardless of the treatment used, the number of *E. coli* did not increase in the first eight days of storage, and treatments with essential oil and extract were considered by the authors to be effective methods of controlling this bacterium.

**Table 1:** Essential oils as antioxidants and/or preservatives applied directly to meat and meat products

Essential oil/species	Major constituent/%	Form of application	Effect	Dose used	Product	Storage condition			Reference
Time/day	*t*/°C	
Sage (*Salvia officinalis*)	-	Direct	AM (*E. coli*)	0.4 and 0.6 µL/g	Minced pork	14	4		(*22*)
Oregano (*Origanum vulgare*) Cinnamon (*Cinnamomum zeylanicum*) Tahiti lemon (*Citrus aurantifolia*) Cardamom (*Elettaria cardamomo*) Chinese pepper (*Litsea cubeba*)	-	Essential oil, emulsions and nanoemulsions	AM (*C. sporogenes*) NR AO	0.2325 and 0.27 %	Mortadella	20	14		(*28*)
Oregano (*Origanum vulgare*)	Carvacrol 77.19	Direct, combined with 0.5 and 1 % radish powder	NR	100 mg/kg	Fermented cooked sausages (pork/beef meat)	30 and 60	4 and 20		(*29*)
Thyme	Thymol 50.48	Direct	AM (*Salmonella* (*S. enteritidis, S.* Typhimurium, *S. montevideo* and *S. infantis*)	0.3, 0.6 and 0.9 %	Pork meat	15		(3±1)	(*30*)
Rosemary (*Rosmarinus officinalis*)	1,8-Cineole 36.2, camphor 16.4	Spraying on packaging	AM (*Pseudomonas* spp., *Brochothrix thermosphacta, Enterobacteriaceae*)	4 %	Beef meat	20 Extended shelf life up to 15 (4 to 5 more days than the control)		4	(*31*)
*Zataria multiflora*,*Origanum vulgare* L., *Satureja bachtiarica*	Carvacrol 35.5, thymol 22, carvacrol 29, γ-terpinene 20, carvacrol 46, thymol 28.5	Direct	AO (*C. perfringens* and *C. sporogenes*) NR	0.355 and 0.71 % 0.395 and 0.79 % 0.275, 0.55 and 1.1 %	Beef meat	30		room	(*32*)

AM=antimicrobial, AO=antioxidant, NR=nitrite reduction

**Table 2:** Plant extracts as antioxidants or preservatives applied directly to meat and meat products

Plant extract/species	Form of application	Effect	Dose used	Product	Storage condition			Reference
Time/day	*t*/°C		
Sage (*Salvia officinalis*)	Direct	AM (*E. coli*)	0.4, 0.6 and 1.0 µL/g	Pork meat	14		4	(*22*)
Olive leaves, green tea stinging nettle	ε-polylysine nanoparticles	AM (*S. aureus, E. coli* and *C. perfringens*) AO, NR	500 ppm (mixed extract)	Sausage	45		4	(*33*)
Pomegranate (*Punica granatum*) peels	Direct	AO AM (aerobic bacteria)	17.25 mg/kg	Sausage	60		4	(*34*)
Oregano (*Origanum vulgare*)	Direct	AO	13.32, 17.79 and 24.01 mL/kg	Lamb burger	120		(-18±1)	(*35*)
*Syzygium antisepticum*	Direct application by dipping into the solution	AM (*S. aureus*)	2, 8 and 32 mg/mL	Cooked chicken	5		4 and 10	(*36*)
Cinnamon, clove, anise	Direct	Reduction of the accumulation of biogenic amines, AO, AM (total aerobic bacterial counts, *Enterobacteriaceae*)	0.3 g/kg	Harbin dry sausage (pork meat)	9		under fermentation	(*37*)
Pomegranate (*Punica granatum*) peels	Direct	AO	0.5 and 1.0 %	Beef meatball	180		(18±1)	(*38*)
Purslane (*Portulaca oleracea*)	Pulverization	AO and AM (*P. aeruginosa*, *B. subtilis* and *B. cereus*)	0.25, 0.50 and 1.0 %	Pork meat	9		4	(*39*)
Olive leaves, green tea, stinging nettle	Direct	Nitrite replacement AO AM (total bacterial count, yeasts and moulds)	500 ppm (mixed extract)	Sausage	45		4	(*40*)
Guarana seed, pitanga leaf	Direct	AO	250 mg/kg	Lamb burger	18		(2±1)	(*41*)

AM=antimicrobial, AO=antioxidant, NR=nitrite reduction

The effect of other essential oils on meat products has also been given in other studies. Ozaki *et al*. (*29*), seeking to reduce nitrite in ‘salaminho’, a product made with pork and beef and fermented during processing, used the essential oil from oregano (100 mg/kg) together with radish powder (0.5 and 1 %). The salamis were stored for 30 and 60 days at 4 and 20 °C, and, despite the sensory acceptance and known activity of oregano essential oil, this oil did not inhibit lipid oxidation and did not show antimicrobial activity at the applied mass fraction. This observation was probably due to the added low mass fraction of oregano and a possible decrease in the concentration of bioactive compounds in its commercial oil (*29*). That study is a good example of the impasse between an effective amount of essential oil for antioxidant and antimicrobial activiity and the sensory acceptance of consumers. This fact is one of the reasons why the application of natural compounds through coatings, films and encapsulation is a better option. On the other hand, Fernandes *et al*. (*35*) observed antioxidant activity when they applied oregano extract directly to lamb hamburger as a possible substitute for the synthetic antioxidant sodium erythorbate and stored it for 120 days at -18 °C. In addition, the treated hamburgers did not differ from those produced with a synthetic antioxidant in terms of sensory acceptance.

Harbin sausage, a dry fermented sausage produced in Harbin (PR China), was evaluated by Sun *et al*. (*37*) after adding cinnamon, clove and anise extracts. The application of the extracts reduced the accumulation of biogenic amines, mainly in the treatment containing cinnamon extract, which inhibited the formation of six of the analyzed amines. The inhibitory effect of the extracts might be related to the inhibition of *Enterobacteriaceae* that can increase the production of biogenic amines such as tyramine, putrescine, cadaverine and histamine. The antimicrobial effect of the extracts is probably due to the synergism of their constituents. In the cinnamon extract, which proved to be the most efficient, the presence of eucalyptol and *trans*-cinnamaldehyde, compounds considered to be antimicrobial, might have cooperated for this effect. Anise extract contains antimicrobial constituents such as carvacrol, linalool, terpineol and eugenol, the last also being present in clove extract. The presence of polyphenols in the spice extracts also contributed to the observed antioxidant activity, which was higher in the anise extract. A major concern when adding extracts to foods is the alteration of sensory characteristics. In this study, in addition to the improved microbiological characteristics in the presence of the spice extracts, the colour and attributes such as flavour, odour, acidity and acceptance received better scores than the control samples.

The use of natural products to replace, even partially, the nitrite preservative has been widely studied. This additive can favour the formation of N-nitrosamines when it reacts with the secondary amines present in the meat. These N-nitrosamines can lead to gastrointestinal cancer (*42*). Thus, the reduction of the use of nitrite in meat products is a factor of interest to researchers, industry and consumers, and the replacement of this preservative by natural compounds was demonstrated to be a good alternative.

Pinelli *et al*. (*28*) evaluated the partial replacement of nitrite by emulsions and nanoemulsions of the essential oils from oregano, lemon, cinnamon, cardamom and pepper in mortadella. Additive or synergistic actions among the components of these oils can be observed when they are mixed. The biological activity of interest increases because of this synergism, which permits the application in lower concentrations with smaller sensory alterations. Although no significant difference in the mean number of *Clostridium sporogenes* spores was observed between the treated samples and the control, the number of *C. sporogenes* cells was lower in the treated samples than in the control. Nitrite (75 ppm) was added to the control and the samples treated with the nanoemulsion of the essential oil mixture. Thus, treatments with an emulsion or nanoemulsion can be alternatives for the control of this microorganism in products such as mortadella because they were more efficient than nitrite itself. In addition to the microbial control, the treatments influenced the residual nitrite and the thiobarbituric acid reactive substances (TBARS) content. The residual nitrite content is expected to decrease during the storage of products made with cured meat, and this decrease indeed occurred. However, the final mass fraction of residual nitrite in mortadella treated with nanoemulsions was significantly higher than in the control, with values higher than 45 mg/kg. It is likely that there were interactions between the oils and nitrite that increased the antimicrobial activity of these treatments. Regarding the TBARS analysis, the lowest values were observed after treatments with emulsions or nanoemulsions. The presence of constituents that have antioxidant characteristics, such as the phenolic compounds present in the oils, leads to known and scientifically proven antioxidant activities. The emulsions and nanoemulsions have been shown to be a good alternative for reducing nitrite in bologna, but the used amounts must still be evaluated to reduce the sensory interference that was still unsatisfactory.

Yuan and Yuk (*36*) applied *Syzygium antisepticum* extract directly to cooked chicken in an attempt to inhibit the growth of *S. aureus*. The highest concentration used, 32 mg/mL, inhibited the growth of the microorganism, but the colour of the meat was altered, a fact that would influence the consumer acceptance. The application of plant extracts and essential oils directly to food products, as already mentioned, can interfere with consumer acceptance because of the changes they cause in the food, such as colour, texture and aroma. For this reason, many researchers opt for application through nanoparticles, nanoemulsions, edible coatings or films.

### Incorporation of essential oils and plant extracts in active packaging applied to meat and meat products: preservative and antioxidant activity

The application of essential oils and plant extracts to meat through packaging has been the main focus of many researchers today because it allows the incorporation of active compounds such as antioxidants and antimicrobials and reduces the likelihood of unpleasant sensory changes for the consumer (*43*). The principal research on the application of essential oils and plant extracts as antioxidants and antimicrobial preservatives incorporated into active packaging and used in meat and meat products is presented in Table 3 (*17*, *44*-*61*) and Table 4 (*20*, *47*, *55*, *62*-*67*). Mehdizadeh *et al*. (*47*) evaluated the conservation of beef packaged with cornstarch and chitosan-based films containing the essential oil from *Thymus kotschyanus* and pomegranate (*Punica granatum*) peel extract. A higher antioxidant and antimicrobial activity was observed of the films with combined essential oils and extract. The film containing oil (2 %) and extract (1 %) inhibited the growth of *Listeria monocytogenes* for 12 days. The effect of the films on the other evaluated microorganisms was also more significant when the oil and the extract were present together. This antimicrobial activity might be related to the principal constituents of the oil, thymol and carvacrol, and to the interactions of phenolic compounds in the extract with sulfhydryl groups of proteins found in bacterial structures.

**Table 3:** Essential oil as antioxidants or preservatives applied to meat and meat products in the form of films and coatings

Essential oil/species	Major constituent/%	Form of application	Effect	Dose used	Product	Storage condition			Reference
Time/day	*t*/°C	
Thyme (*Thymus vulgaris* L.)	Thymol 47	Sodium alginate-based films with micro- and nanoemulsions	ATM (coliforms, *S. aureus*, lactic acid bacteria, moulds and yeasts)	0.05 and 00.04 %	Ground meat	8		(4.0±1)	(*44*)
Lemon verbena (*Aloysia citriodora*) Clove (*Syzygium aromati*)	Eugenol 14.63 d-Limonene 12.41 Eugenol 79.4	Sodium alginate-based coatings with and without modified atmosphere	AM (total bacterial count, *Pseudomonas*, lactic acid bacteria, psychrotrophic bacteria, *Enterobacteriaceae*, moulds and yeasts) AO	0.2 and 0.5 %	Chicken breast	15		refrigerated	(*17*)
Rosemary (*Rosmarinus officinalis*)	1,8-cineole 27.52 α-pinene 21.15	Whey protein isolate-based film	AM (total count of psychrotrophic bacteria) AO	2 %	Lamb meat	15		(4.0±1)	(*45*)
*Zataria multiflora*	Thymol 37.94	Corn starch films	AO	6 %	Ground beef patties	20		(4.0±1)	(*46*)
*Thymus kotschyanus*	Thymol 26.61 Carvacrol 12.60	Films based on corn starch and chitosan	AM (*Pseudomona*s, lactic acid bacteria and *L. monocytogenes*) AO	1 and 2 %	Beef	21		(4.0±1)	(*47*)
Star anise (*Illicium verum*)	-	Coating based on soy protein isolate and lectin with nisin and polylysin	AM (viable aerobic bacteria and *E. coli*)	0.4 and 0.6 %	Yao meat	20		(4.0±1)	(*48*)
Cumin (*Cuminum cyminum*)	-	Chitosan-based coating	AM (total count of bacteria, *Enterobacteriaceae*, *S. aureus*, *E. coli*, mold and yeasts), AO	0.2, 0.4 and 0.6 %	Chicken meat	9		4.0	(*49*)
Black cumin (*Nigella sativa*)	-	Multilayer film based on chitosan and alginate	ATM (*S. aureus* and *E. coli*) ANT	1 %	Chicken meat	5		4.0	(*50*)
*Ziziphora persica*	Pulegone 31.42, Neomenthol 18.58	Alginate based coating	AM (*E. coli, S.* Typhimurium, *P. aeruginosa, L. monocytogenes, B. cereus, S. aureus*) AO	0.5 and 1 %	Chicken meat	12		4.0	(*51*)
Rosemary (*Rosmarinus officinalis*)	-	Chitosan-based film	AM (mesophilic aerobic bacteria, *B. cereus, S. aureus, L. monocytogenes, S. enterica, E. coli, C.albicans*) AO	0.5, 1.0 and 2.0 %	Chicken meat	15		(5.0±2)	(*52*)
Rosemary (*Rosmarinus officinalis*)	-	Nanogel encapsulation of benzoic acid and chitosan applied as coating	AM (*S. typhimurium*)	0.5, 1.0 and 2.0 mg of nanoencapsulated oil per g of meat	Beef cutlet	12		4.0	(*53*)
Oregano (*Origanum vulgare*)	-	Direct and nanoemulsion encapsulation	AM (*S. aureus* and *E. coli*)	5 %	Chicken pate	8		(4.0±2)	(*54*)
*Zataria multiflora*	-	Chitosan-based coating with Sumac extract	AO and AM (total mesophilic bacteria, lactic acid bacteria, *Enterobacteriaceae*, *Pseudomonas*, fungi and yeasts)	1 %	Meat	20		4.0	(*55*)
Satureja (*Satureja khuzestanica*)	-	Chitosan-based coating	AO and AM (*Pseudomonas*, total count of bacteria and lactic acid bacteria)	1 %	Lamb meat	20		4.0	(*56*)
Oregano	-	Pectin-based coating with resveratrol nanoemulsion	AO, AM (total bacterial count)	0.5 %	Pork loin	20		4.0	(*57*)
*Z. multiflora*	-	Chitosan and gelatin-based nanofibers	NR AM (*C. perfringens*)	20 and 40 %	Sausage	20		(4.0±1)	(*58*)
Rosemary (*Rosmarinus officinalis*) Ginger (*Zingiber officinale*)	-	Chitosan-based films	AO	2 %	Chicken meat	15		(5.0±2)	(*59*)
Cinnamon	-	Polylactide films plasticized with Ag-Cu nanoparticles	AM (*S.* Typhimurium, *C. jejuni* and *L. monocytogenes*)	25 and 50 %	Chicken meat	21		4.0	(*60*)
Ajowan (*Trachyspermum ammi*)	Thymol 70.95	Films based on gelatin and carboxymethylcellulose with chitin nanofiber	ATM (total viable count, psychotrophic count, *Pseudomonas* spp., *S. aureus*, lactic acid bacteria, moulds and yeasts)	0.24, 0.64 and 1 %	Beef	15		4.0	(*61*)

AM=antimicrobial, AO=antioxidant, NR=nitrite reduction

**Table 4:** Plant extracts as antioxidants or preservatives applied to meat and meat products in the form of films and coatings

Plant extract/species	Form of application	Effect	Dose used/%	Product	Storage condition		Reference
Time/day	*t*/°C	
Pomegranate (*Punica granatum*) peel	Films based on corn starch and chitosan	AO AM (*Pseudomona*s spp., lactic acid bacteria and *L. monocytogenes*)	0.5 and 1	Beef	21	(4.0±1)	(*47*)
Red cabbage	Films based on starch and whey	AO SP	64.18 and 50	Ground beef	4	4.0	(*62*)
Laurel (*Laurus nobilis* L.), sage (*Salvia officinalis*)	Whey protein isolate based films	AO	2 and 4	Cooked meatballs	60	-18.0	(*63*)
Sumac (*Rhus coriaria*)	Chitosan-based coating with essential oil from *Zataria multiflora*	AO and AM (total mesophilic bacteria, lactic acid bacteria, *Enterobacteriaceae*, *Pseudomonas*, fungi and yeasts)	2 and 4	Beef	20	4.0	(*55*)
Shatavari (*Asparagus racemosus*)	Edible film based on calcium alginate and maltodextrin	AO and AM (total bacterial count, and yeast and mould counts)	1 and 2	Salsage	21	(4.0±1)	(*64*)
Stinging nettle (*Urtica dioica*)	ε-polylysine coating	AO, AM (moulds and yeasts, total bacterial and coliform counts)	3, 6 and 9	Beef	12	4.0	(*65*)
Grape seed	Chitosan/gelatin-based coating	AO	0.5	Pork	20	4.0	(*66*)
Green tea	Organic film	AO	6 and 8	Pork	14	4.0	(*67*)
Saffron leaves	Films based on chitosan and methylcellulose nanofiber	AM (*E. coli* and *S. aureus*) AO SP	3	Lamb meat	3	25.0	(*20*)

AM=antimicrobial, AO=antioxidant, SP=smart packaging

Langroodi *et al*. (*55*) also evaluated the application of a combination of essential oils and extracts to beef. The results of the application of chitosan-based coatings with 1 % essential oil from *Zataria multiflora* and *Rhus coriaria* extract (2 and 4 %) showed that both the extract and the essential oil contributed to the antioxidant activity of the coatings, yielding significantly lower TBARS and peroxide values. The microbial activity was the lowest at the highest concentration of the extract, and the microbiological quality of all the samples was maintained for 20 days. On the other hand, the quality of the control samples was lost after the fifth day of storage. Therefore, an additive or synergistic effect against the evaluated microorganisms was observed when using the combination of the extract with the essential oil.

The ground beef product that undergoes minimal processing can be used for other products such as hamburgers and meatballs. This product has been evaluated in various studies that applied the oils and extracts to determine the antioxidant and preservative activity of these natural compounds. Almasi *et al*. (*44*) developed films based on sodium alginate containing the essential oil from *Thymus vulgaris* to determine their antimicrobial activity on ground beef. These authors applied 0.05 and 0.04 % of the oil, respectively, using two different techniques, microemulsion and nanoemulsion, and they evaluated the antimicrobial activity of ground beef in contact with the film and under refrigeration. A significant antimicrobial activity against all the tested microorganisms was observed with the films made by the microemulsion technique, with the emphasis on the number of total mesophiles for which a decrease of 2 logarithmic cycles (100 times) relative to the control was found after eight days of storage. This activity is explained by the greater availability of the essential oil that comes into contact with the meat product when it is present in a microemulsion. In addition, the particles diffuse through the films more easily, which makes the oils more readily available to interfere with the cellular activities of microorganisms. The surfactant micelles formed in the films can fuse with the phospholipid bilayers that make up the cell membrane to increase the interaction with bacterial cells. This interaction thereby increases the antimicrobial activity, which can lead to cell death.

Work by Akcan *et al.* (*63*) showed that interesting results were also obtained with meat products made from ground beef, such as meatballs and hamburgers. Films based on isolated whey proteins containing extracts of *Laurus nobilis* or *Salvia officinalis* were applied to cooked meatballs. Antioxidant activity throughout storage was observed in the presence of the films, but research to improve the sensory acceptability of the product is necessary. Subsequently, Amiri *et al*. (*46*) investigated the application of cornstarch-based films made by a nanoemulsion containing essential oils from *Zataria multiflora* and applied to hamburger steaks. The increase in pH during storage was lower with the films containing essential oils, and the oxidation of protein and lipid was also lower, especially with the nanoemulsions. The oxidative stability increased with the use of smaller nanoemulsion droplets. The product of that study was sensorially well accepted, but there was a decrease in the acceptability during the days of storage, whereas the control was unacceptable from the tenth day onwards.

Good results were also obtained when the red cabbage extract was incorporated into films based on starch and whey and applied to ground beef. Sanches *et al*. (*62*) observed that the films acted as antioxidants, especially at a amount of 64.15 %, which was sufficient to stabilize oxymyoglobin. This bright red pigment is a derivative of myoglobin, one of the main pigments responsible for meat colour (*2*). Sanches *et al*. (*62*) attributed the high concentration of anthocyanins present in the extract to the antioxidant activity of this film. In addition to helping to preserve the characteristics of the meat, the film possessed the ability to monitor the quality of the product through its colour change due to the change of pH value, and it was thus characterized as a smart packaging. According to the authors, the change in the colour of the film occurred as a result of the colour change of the present anthocyanins. Anthocyanins are red or purple (due to the flavylium cation) at low pH, but at high pH, they turn blue (formation of quinoidal bases). If the pH continues to increase, the sample becomes colourless (formation of chalcones). High pH values in meat are indicative of microbial spoilage and protein degradation. Therefore, this type of packaging can indicate when the meat is unfit for consumption.

Smart packaging has also been designed for application to lamb meat (*20*). The film obtained from chitosan and methylcellulose nanofiber was incorporated with anthocyanin extract from saffron leaves. The extract was applied to meat that was stored for three days at 25 °C. The anthocyanins present in the extract were responsible for changing the colour of the film by altering the pH of the meat, which indicated the presence of deterioration. In addition to the indication of quality, the films indicated that antimicrobial and antioxidant activity existed, but these biological activities were not evaluated in the meat.

Lamb meat was also evaluated using films embedded with the essential oil from *Rosmarinus officinalis* and coatings embedded with the essential oil from *Satureja khuzestanica* (*45*). The films with rosemary essential oil (2 %) were made from whey proteins and had antioxidant and antimicrobial activities. The addition of rosemary oil was efficient to the point of extending the shelf life of the product from about six days to 12 to 15 days.

The coatings studied by Alizadeh-Sani *et al.* (*45*) were made with chitosan and savory essential oil (1 %) and had sufficient antioxidant and antimicrobial activities to exceed the recommended microbiological limit (7 log CFU/g) only after 20 days in the treated samples, whereas the control exceeded this limit after nine days of storage. Previously, Pabast *et al*. (*56*) studied the application of chitosan-based coatings and concluded that, even without the addition of essential oils, these coatings were able to reduce the pH and act as antimicrobial agents.

The projections of world consumption and production of chicken breast have increased in recent years (*49*). Several studies on the application of natural compounds to chicken meat have been performed. Hosseini *et al*. (*17*) studied the effect of adding the essential oil from *Aloysia citriodora* and *Syzygium aromaticum* to chicken breasts in the form of coatings. Sodium alginate-based coatings were made with each oil and the combination of the oils. Antioxidant and antimicrobial activities were observed of the oils, and the shelf life of the product increased. The use of a modified atmosphere increased the antibacterial effect, and the best effect was observed in the application of the coating containing two oils at 0.5 % each. No significant difference between the treatments was observed in the sensory analysis. Good results were also observed with other essential oils, such as those of *Cuminum cyminum* (*50*), *Nigella sativa* (*51*) and *Ziziphora persica* (*52*), which were applied to chicken meat through coatings and films, and preserved the meat stored at 4 °C for 9, 5 and 12 days, respectively.

Satisfactory results were obtained with the essential oil from *Rosmarinus officinalis* when it was incorporated into coatings and applied to chicken breasts (*57*). Because rosemary is a condiment commonly used in meat products in its natural form, consumers tend to recognize the odour and flavour of this plant and do not reject it in coated meat. Thus, the sensory evaluation of the product does not tend to have negative results. Films made from chitosan with rosemary oil were applied to chicken meat, and antioxidant and antimicrobial activities were measured. The total counts of mesophilic aerobic bacteria were lower in the samples treated with the active films. According to the authors, the antimicrobial activity of the films was related to chitosan, and the presence of phenolic compounds derived from rosemary essential oil increased the shelf life of the product. The control sample from the third day onwards was rejected. Thus, that study emerged as a new way to complement the necessary daily consumption of phenolic compounds.

Oregano is also a condiment widely used in food preparation. In addition to presenting biological activities of interest, it is able to improve the quality when applied to meat, mainly because of the action of its principal compounds, thymol and carvacrol, which are efficient inhibitors of bacterial growth. Xiong *et al*. (*67*) applied oregano essential oil incorporated into pectin-based coatings containing a resveratrol nanoemulsion to pork. The meat was stored for 20 days at 4 °C, whereas the total bacterial count in the control sample was considered microbiologically unacceptable from day 15 onwards, exceeding 7 log CFU/g. The treated samples remained below the limit during the 20 days of storage. Furthermore, lipid oxidation was lower with the treatments, whereas the limit of malondialdehye of 0.5 mg/kg was exceeded in the control on the fifth day. The authors concluded that the essential oil from oregano and resveratrol can scavenge free radicals and stop oxidation chain reactions.

The antioxidant activity in pork was reported by Song *et al*. (*68*), who observed lower TBARS values of the treated meat during storage than of the control when films containing green tea extract were applied. It was also observed that the changes in the TBARS values were insignificant in the extract-treated samples during storage.

## LIMITATIONS OF THE APPLICATION OF ESSENTIAL OILS AND PLANT EXTRACTS IN MEAT AND MEAT PRODUCTS

The biological activity of essential oils and plant extracts is increasingly known among researchers, consumers and industries. The existing demand for healthy products can be met using these natural compounds of low toxicity (*4*).

The application of extracts and essential oils to meat and meat products as antioxidant and antimicrobial agents yields excellent results, as was presented in the previous chapters. However, the application of these natural compounds to food still faces some technological challenges. According to Silva *et al*. (*4*), the complexity of the composition of meat-based foods, such as amounts of proteins, lipids and moisture, among others, leads to the interaction of natural compounds with other components of the food, and thus, they are less readily available to act on microorganisms. Other properties, such as water activity and pH, can also influence the performance of natural compounds. Thus, food applications can require concentrations up to 100 times greater than those used in in vitro experiments.

The first point to observe for the application of a compound in food that will be offered to consumers is its safety. Despite being completely natural, some essential oils and plant extracts can be unsuitable for consumption in certain concentrations. Another important point to be observed is the form of application of the compounds. Despite the biological properties already described, the fact that they have a striking characteristic aroma and flavour makes their application in food difficult. To facilitate this application, the incorporation into edible, biodegradable coatings and films made with biopolymers are an alternative for the preservation of food. Thus, it is possible to obtain a material with the activity of interest while improving the value of the food (*43*, *69*).

The direct use of natural compounds in meat and meat products, as mentioned, can completely change the sensory characteristics of the product, and it might not be very acceptable to consumers, which limits its application (*70*). Danilović *et al*. (*22*) emphasized the fact that the oils and extracts can cause changes in odour and flavour, and therefore, they should be used in the lowest possible concentration. However, the concentration must be sufficient for the action of interest: antioxidant or antimicrobial activity, increase in the shelf life of the product, among others. According to Moraes-Lovison *et al*. (*54*), this challenge can be overcome by using encapsulation and nanoemulsification techniques for the application of natural compounds. These alternative applications of essential oils and plant extracts in meat and meat products can be presented as an economically viable industrial alternative. These, in addition to the advantages already mentioned throughout the text, are low cost, depending on the polymers and plant materials used in the process, easy production and, in general, they do not require high equipment costs (*71*, *72*).

## CONCLUSIONS

Good results were obtained with essential oils and plant extracts when they were applied to beef, pork, goat and poultry. They acted by preserving the products, and consequently, increasing their shelf life. Antimicrobial and antioxidant activities of the extracts and essential oils were observed, and they are possible substitutes for synthetic additives. Many studies have suggested the application methods that have a lower impact on the sensory characteristics of meat products, such as application in films, coatings, emulsions and nanoemulsions. However, studies aimed at alternatives for the application of these natural compounds with the objective of impacting the sensory quality of the products as little as possible must still be explored.
